# Electrospun Carbon Nanofiber–TiN Composite Interlayers for Improved Cycling Stability and Rate Capability of Lithium–Sulfur Batteries

**DOI:** 10.3390/molecules31203628

**Published:** 2026-10-12

**Authors:** Chaoqun Niu, Huan Liu, Zisheng Chao

**Affiliations:** 1 School of Physics and Electronic Science, Changsha University of Science & Technology, Changsha 410114, China; chaoqunniu@yeah.net; 2 Key Laboratory of Energy Conversion Photoelectronic Functional Materials of Jiangxi Education Institutes, School of Mathematics and Physics, Jinggangshan University, Ji’an 343009, China; 3 Shinawatra University, 99, Bang Toei, Sam Khok District, Pathum Thani 12160, Thailand; 4 School of Materials Science and Engineering, Changsha University of Science & Technology, Changsha 410114, China

**Keywords:** lithium-sulfur batteries, titanium nitride, electrospinning, functional interlayer, polysulfides

## Abstract

Lithium–sulfur (Li-S) batteries offer a high theoretical energy density of 2600 Wh·kg^−1^ and a theoretical specific capacity of 1675 mAh·g^−1^; however, their practical application is severely hindered by the shuttle effect of soluble lithium polysulfides, the intrinsic electronic insulating nature of sulfur and Li_2_S, and the substantial volume variation during charge/discharge processes. In this study, titanium nitride (TiN) powder was first prepared, and a carbon nanofiber/titanium nitride (CNF@TiN) composite nanofiber membrane was subsequently fabricated by electrospinning followed by pre-oxidation/carbonization treatment and employed as a functional interlayer between the separator and sulfur cathode. X-ray diffraction (XRD) and high-resolution transmission electron microscopy (HRTEM) showed that the obtained TiN possessed a well-crystallized face-centered cubic structure. Scanning electron microscopy (SEM), transmission electron microscopy (TEM), and energy-dispersive X-ray spectroscopy (EDS) revealed that TiN particles were relatively uniformly dispersed and anchored within the three-dimensional interconnected CNF network. X-ray photoelectron spectroscopy (XPS) analyses of the composite and the TiN powder confirmed the presence of Ti–N bonding, revealed N/O-containing functional groups on the carbon scaffold, and indicated only a thin oxidized layer on the TiN particle surface. Nitrogen adsorption–desorption analysis confirmed the porous character of the fibrous interlayers. Electrochemical tests showed that the cell equipped with the CNF@TiN interlayer delivered an initial discharge specific capacity of 1205 mAh·g^−1^ at 0.1 C and retained a reversible capacity of approximately 1010 mAh·g^−1^ after 100 cycles. At 0.2 C, the initial discharge specific capacity was approximately 1065 mAh·g^−1^, and approximately 875 mAh·g^−1^ was retained after 300 cycles, corresponding to a capacity retention of 82.2% and an average capacity decay of approximately 0.059% per cycle. Rate capability, cyclic voltammetry (CV), electrochemical impedance spectroscopy (EIS), and galvanostatic charge–discharge measurements indicated that the CNF@TiN interlayer favors reversible sulfur-species conversion and improved interfacial reaction kinetics. The enhanced performance can be attributed to the physical confinement, electron-transport, and structural-support functions of the CNF network, together with the polar TiN interface, whose presence is consistent with restricted polysulfide migration and facilitated interfacial conversion. This work provides a feasible strategy for designing multifunctional interlayers integrating electrical conductivity, physical confinement, and interfacial regulation for Li-S batteries.

## 1. Introduction

Lithium–sulfur (Li-S) batteries are considered one of the most promising next-generation high-specific-energy rechargeable battery systems because of their high theoretical energy density (2600 Wh·kg^−1^) and theoretical specific capacity (1675 mAh·g^−1^) [1,2,3]. Compared with conventional cathode materials for lithium-ion batteries, elemental sulfur offers the advantages of natural abundance, relatively low cost, and environmental friendliness and has therefore attracted extensive attention for electric transportation and large-scale energy-storage applications [4,5,6,7,8]. Nevertheless, the practical deployment of Li-S batteries remains constrained by several key issues. First, elemental sulfur and its fully discharged product Li_2_S both exhibit low electronic conductivity, which limits electrode reaction kinetics [9,10,11]. Second, soluble long-chain lithium polysulfides generated during charge/discharge can migrate between the cathode and anode, causing the characteristic “shuttle effect”, which leads to active-material loss, reduced Coulombic efficiency, and parasitic reactions at the lithium-metal anode [12,13,14,15]. In addition, the pronounced density difference between sulfur and Li_2_S results in considerable volume variation during cycling, further compromising electrode structural integrity and cycle life [16].

To address these challenges, extensive material-design strategies have been explored, including sulfur-host engineering, separator modification, and the introduction of functional interlayers [17,18,19]. Carbon-based materials such as porous carbon, carbon nanotubes, and graphene possess good electrical conductivity and porous structures and can retard polysulfide diffusion through physical confinement; however, their nonpolar surfaces generally interact weakly with polar lithium polysulfides [20,21,22]. In contrast, polar materials, including transition-metal oxides, sulfides, and nitrides, can strengthen interfacial interactions with polysulfides [23,24,25]. Among them, titanium nitride (TiN) combines metallic-like conductivity, good chemical stability, and strong affinity toward polysulfides [26,27]. The surface Ti active sites can interact strongly with sulfur species, while its relatively high electronic conductivity is favorable for reducing interfacial polarization and facilitating polysulfide redox conversion [28,29]. Therefore, coupling TiN with a continuous carbon network is expected to integrate polysulfide confinement, interfacial regulation, and efficient electron transport [30].

Previous studies have demonstrated the potential of TiN-based architectures for Li–S batteries. Mesoporous TiN has been shown to enable highly stable cycling through chemisorption of polysulfides [17]; TiN-based membranes have been reported to act as reactor-like interlayers that regulate both S8/Li_2_S conversion and lithium-dendrite growth [15]; and hollow multishelled TiN structures have served as multifunctional catalytic hosts [8]. TiN/carbon composites, such as coaxial CNTs@TiN–TiO_2_ sponges [27] and TiN–VN heterostructures embedded in conductive frameworks [28], further combine the adsorption and catalytic functions of TiN with the conductivity of carbon, while two-dimensional TiN nanosheets [29] and graphene-like porous carbon–TiN composites [19] have been exploited as separator coatings. Despite these advances, several limitations remain: many TiN/carbon hybrids require multi-step or template-assisted syntheses; TiN particles are frequently aggregated or only partially exposed, which limits the accessible polar interface; and the long-term structural stability of free-standing interlayers under repeated cycling is rarely addressed. Electrospinning offers a facile and scalable route to free-standing, interwoven carbon nanofiber networks; however, electrospun carbon interlayers decorated with uniformly dispersed polar TiN particles—which could integrate physical confinement, continuous electron transport, and polar interfacial regulation within a single self-standing membrane—have been rarely reported [26,30]. Motivated by this gap, the present work combines the electrospinning strategy with pre-prepared TiN particles to construct such a self-standing CNF@TiN composite interlayer, as detailed below. It should be emphasized that the strong polysulfide affinity of TiN [17] and the general concept of combining electrospun carbon nanofibers with polar titanium-based compounds [30] are established background. The novelty of the present work therefore lies not in these concepts themselves, but in the specific architecture—pre-synthesized, well-crystallized TiN particles uniformly anchored within a self-standing, N/O-functionalized PAN-derived carbon nanofiber network through a single, straightforward electrospinning–carbonization route—and in the systematic correlation of its surface chemistry and interfacial structure with the electrochemical behavior.

Electrospinning enables the construction of continuous, interconnected, and highly porous one-dimensional fibrous networks, providing a convenient route for fabricating self-supporting functional interlayers. Accordingly, in this study, TiN powder was first prepared and dispersed in a polyacrylonitrile (PAN) spinning precursor solution. A carbon nanofiber/titanium nitride (CNF@TiN) composite nanofiber membrane was then obtained through electrospinning, pre-oxidation, and high-temperature carbonization and placed between the separator and a sulfur/carbon nanotube (S/CNT) cathode as a functional interlayer. The crystal structure, surface chemical states, and microstructure of the composite were characterized by X-ray diffraction (XRD), X-ray photoelectron spectroscopy (XPS), scanning electron microscopy (SEM), transmission electron microscopy (TEM), and energy-dispersive X-ray spectroscopy (EDS). Its electrochemical performance was evaluated by rate capability, cycling tests, cyclic voltammetry (CV), electrochemical impedance spectroscopy (EIS), and galvanostatic charge–discharge measurements. The study focuses on the complementary functions of the three-dimensional conductive CNF network and the polar TiN interface in relation to reaction reversibility and cycling stability in Li-S batteries. Throughout this work, physical confinement (retardation of polysulfide diffusion by the fibrous network), chemical adsorption (polar interactions at the TiN surface), and conversion kinetics (the rates of the sulfur redox reactions) are treated as related but distinct phenomena.

## 2. Results and Discussion

**Figure 1:**
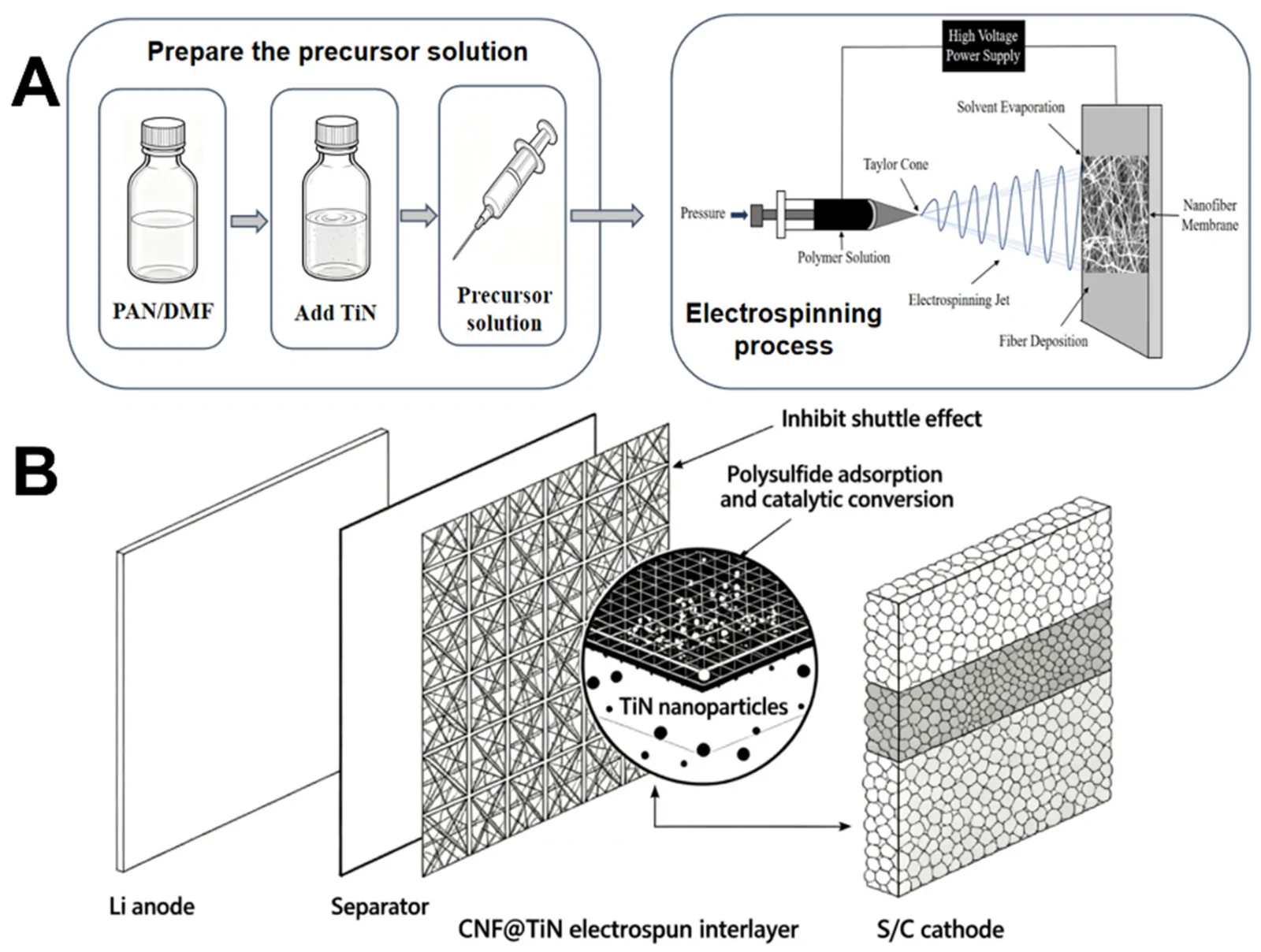
(**A**) Schematic illustration of the fabrication process for the CNF@TiN composite nanofiber interlayer; (**B**) schematic configuration of a lithium–sulfur battery containing the CNF@TiN functional interlayer.

Figure 1A schematically illustrates the fabrication process of the CNF@TiN composite nanofiber interlayer. PAN was first dissolved in DMF, followed by the addition and thorough dispersion of pre-prepared TiN particles to obtain a PAN/TiN spinning precursor solution. A PAN/TiN nanofiber membrane was then produced by electrospinning. After drying, pre-oxidation, and high-temperature carbonization under an inert atmosphere, a CNF@TiN composite membrane with TiN particles supported within the carbon nanofiber network was obtained and cut to the required size for use as a functional interlayer in Li-S batteries.

Figure 1B shows the configuration of a Li-S battery containing the CNF@TiN functional interlayer, consisting of a lithium-metal anode, a polypropylene separator, a CNF@TiN composite nanofiber interlayer, and an S/CNT cathode. The CNF@TiN interlayer is positioned between the separator and cathode. Its three-dimensional interwoven carbon nanofiber network can physically hinder the diffusion of soluble polysulfides while providing continuous electron-transport pathways. Meanwhile, the polar TiN particles dispersed throughout the fibrous network can enhance interfacial adsorption of polysulfides and facilitate their redox conversion, thereby acting through complementary functions to mitigate the shuttle effect and improve active-material utilization and cycling stability.

### 2.1. Structure and Morphology

**Figure 2:**
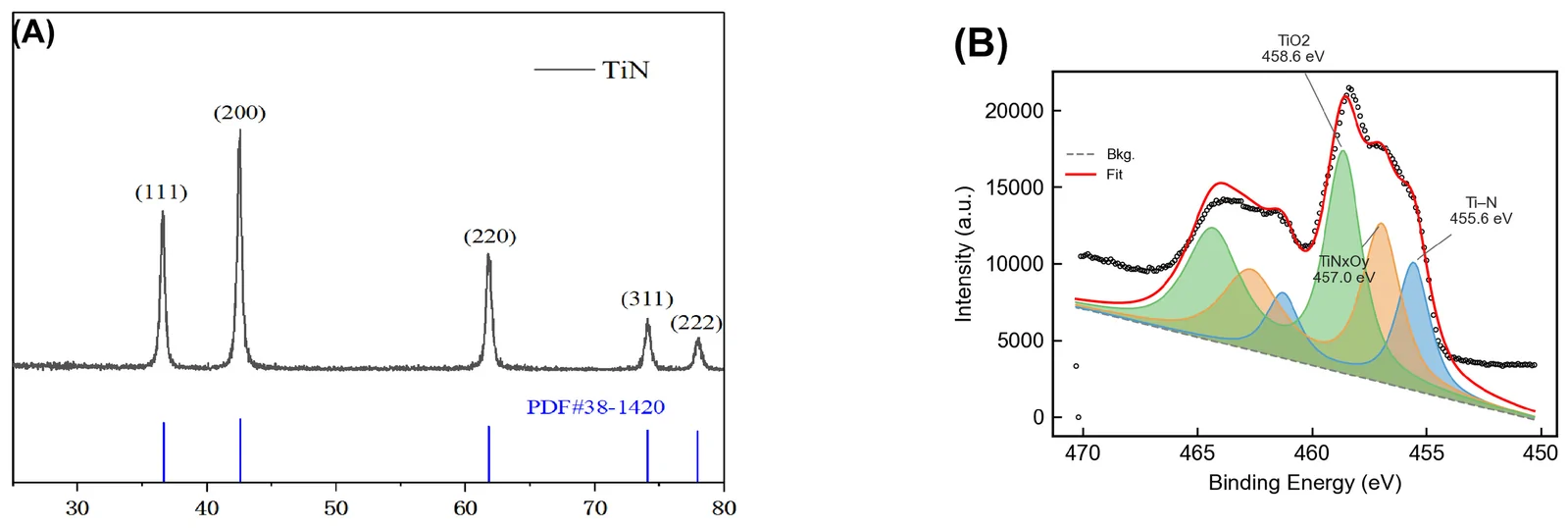
(**A**) XRD pattern of the prepared TiN powder; (**B**) high-resolution Ti 2p XPS spectrum of the CNF@TiN composite interlayer.

Figure 2A shows the X-ray diffraction (XRD) pattern of TiN powder obtained by high-temperature nitridation of the TiO_2_ precursor under NH3. In the collected pattern (2θ = 10–80°), five major diffraction peaks are observed at approximately 36.7°, 42.6°, 61.8°, 74.1°, and 78.0°, which can be indexed to the (111), (200), (220), (311), and (222) planes of face-centered cubic TiN (space group Fm-3m), respectively, in good agreement with the standard Powder Diffraction File (JCPDS No. 38-1420) and previously reported XRD patterns of rock-salt TiN [8,17,27]. The diffraction peaks are relatively sharp, and no obvious impurity-phase peaks are detected within the measured range, indicating that the obtained product is predominantly a well-crystallized TiN phase. The high crystallinity of TiN is beneficial for maintaining metallic-like electronic conductivity and stable polar adsorption sites during cell operation [17,28]. It should be noted that the minor TiNxOy/TiO_2_-type surface species detected by XPS (Figure 2B) are confined to a thin surface layer and are below the detection limit of laboratory XRD; their presence at trace levels therefore cannot be excluded from the diffraction data alone.

Figure 2B presents the high-resolution Ti 2p XPS spectrum of the CNF@TiN composite. After charge correction against the adventitious C 1s peak at 284.8 eV, peak deconvolution yields a Ti 2p3/2 component at approximately 455.6 eV, which is assigned to Ti–N bonding in titanium nitride [31], consistent with the values reported for TiN-based Li–S battery materials [8,15,27]. The component at approximately 457.0 eV is attributed to titanium oxynitride (TiNxOy)-type surface species, and that at approximately 458.6 eV to TiO_2_, both arising from slight surface oxidation of the TiN particles upon air exposure [31,32]. Because the binding energies of Ti-containing species vary with the chemical environment and surface oxidation state, the distinction between TiNxOy and TiO_2_ is based on the following considerations: (i) the two components are separated by approximately 1.6 eV, which is well beyond the typical experimental uncertainty (±0.1–0.2 eV); (ii) their binding energies match the ranges reported for titanium oxynitrides and TiO_2_, respectively [31,32]; and (iii) upon Ar+ sputter etching of the TiN powder for 430 s (Figure S2), the Ti–N fraction increases markedly (from approximately 20% to 41% of the Ti 2p envelope) while the TiO_2_ fraction decreases (from approximately 46% to 30%), directly confirming that the oxidized species are confined to a thin surface layer above the nitride core rather than extending into the bulk. Overall, these XPS results are consistent with the presence of TiN in the composite accompanied by slight surface oxidation, in agreement with the XRD and HRTEM results discussed above. The relative integrated contributions of the Ti–N, TiNxOy, and TiO_2_ components are approximately 20%, 34%, and 46%, respectively.

The CNF@TiN composite interlayer was further characterized by high-resolution XPS in the C 1s, N 1s, O 1s, and Ti 2p regions (Figure S1). The C 1s spectrum (Figure S1a) is dominated by the sp2-hybridized graphitic C=C component at 284.8 eV, accompanied by a C–N/C–O component at approximately 286.5 eV, a C=O component at approximately 288.9 eV, and a π–π* shake-up feature at approximately 291.5 eV, reflecting the N/O-functionalized, moderately defective surface of the PAN-derived carbon scaffold [33,34]. The N 1s spectrum (Figure S1b) contains a nitride component at approximately 396.6 eV, characteristic of Ti–N bonding [30,31,32], together with an oxynitride-type component at approximately 398.4 eV and a component at approximately 400.5 eV assignable to pyridinic and pyrrolic nitrogen configurations retained from the PAN precursor in the carbon scaffold [34]. The O 1s spectrum (Figure S1c) can be deconvoluted into a lattice-oxygen component associated with titanium oxide species (~530.0 eV), an intermediate component (~531.1 eV) assigned to titanium oxynitride species and surface hydroxyl groups, and a minor component (~534.2 eV) from adsorbed water and other oxygen-containing species [31,32]. The Ti 2p spectrum (Figure S1d) is consistent with that shown in Figure 2B, comprising Ti–N, TiNxOy, and TiO_2_-type components. It should be emphasized that XPS is a surface-sensitive technique with a probe depth of only a few nanometers; the prominence of the oxidized Ti components and the O 1s signal therefore mainly reflects a thin surface oxynitride/oxide layer formed upon exposure of the TiN particles to air, together with adsorbed oxygen-containing species, rather than bulk oxidation of the nitride [31]. These results indicate that the carbon scaffold is an N/O-functionalized carbon network and that the embedded TiN particles carry polar Ti–N sites beneath a thin surface-oxidized layer, both of which are expected to be favorable for interaction with polysulfide species.

The as-prepared TiN powder was additionally characterized by XPS to trace possible impurities and to probe the composition beneath the native surface oxide layer (Figure S2). To this end, the powder surface was sputter-etched with Ar^+^ ions for 430 s before data acquisition. The survey spectrum of the etched powder (Figure S2a) contains only Ti, N, O, and a small amount of residual carbon; within the detection limit of XPS, no signals from other elements—such as residual Na that could originate from the alkaline treatment step of the synthesis—were detected. The high-resolution Ti 2p spectrum of the etched powder (Figure S2b) contains the same Ti–N, TiNxOy, and TiO_2_ components observed for the composite (see Figure 2B); however, the Ti–N contribution increases from approximately 20% to 41% of the Ti 2p envelope while the TiO_2_ contribution drops from approximately 46% to 30%, confirming that the oxidized species are confined to a thin surface layer that is largely removed by etching. The N 1s spectrum (Figure S2c) is dominated by the nitride component at approximately 397.0 eV [30,31,32], and the O 1s intensity (Figure S2d) decreases correspondingly, consistent with a thin surface-oxidized layer and adsorbed oxygen-containing species rather than a bulk oxide [31,32]. These results confirm that the nitridation product is TiN carrying a thin surface oxynitride/oxide layer formed upon air exposure, in agreement with the XRD results (Figure 2A).

**Figure 3:**
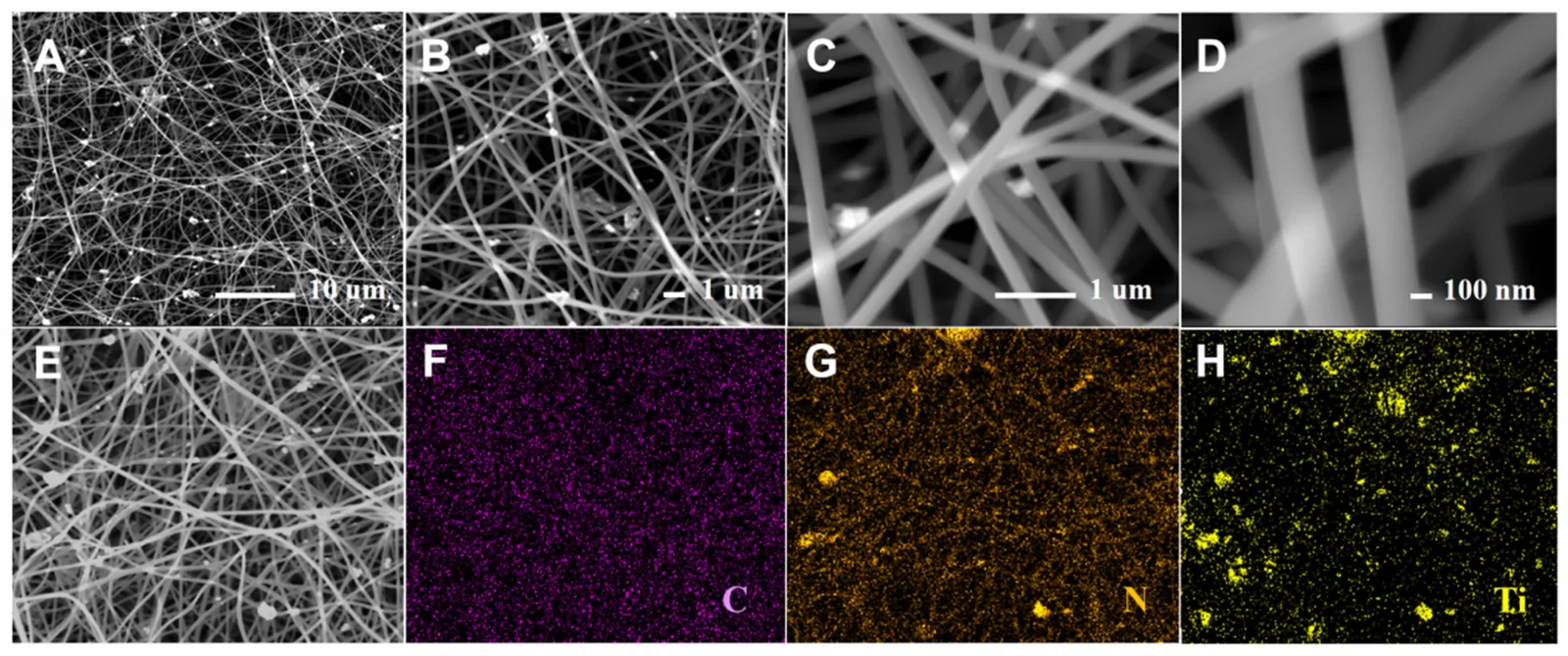
(**A**) Low-magnification SEM overview of the CNF@TiN interlayer; (**B**) SEM image of the interwoven nanofiber network; (**C**) SEM image of individual composite nanofibers; (**D**) high-magnification SEM image of TiN particles anchored on the nanofibers; (**E**) SEM image of the region selected for EDS analysis; (**F**) EDS elemental map of C; (**G**) EDS elemental map of N; (**H**) EDS elemental map of Ti.

Figure 3 shows the SEM morphology and EDS elemental distributions of the CNF@TiN composite nanofiber interlayer. As shown in Figure 3A–D, the electrospun nanofibers are interwoven to form a continuous three-dimensional network. The fibers exhibit a relatively uniform morphology without obvious large-scale fracture or severe aggregation. Particulate features can be observed on the fiber surfaces; combined with the EDS results discussed below, these features are attributed to TiN particles anchored on the carbon nanofibers. This porous fibrous network provides space for electrolyte wetting and ion transport while establishing continuous pathways for electron conduction, a function similarly reported for other electrospun carbon interlayers [20,22,26]. Figure 3E shows the selected SEM region, and Figure 3F–H present the corresponding elemental maps of C, N, and Ti. The elemental signals exhibit good spatial correspondence with the fiber/particle morphology, indicating that the TiN component was successfully incorporated into the CNF framework and remained relatively uniformly distributed throughout the composite membrane.

**Figure 4:**
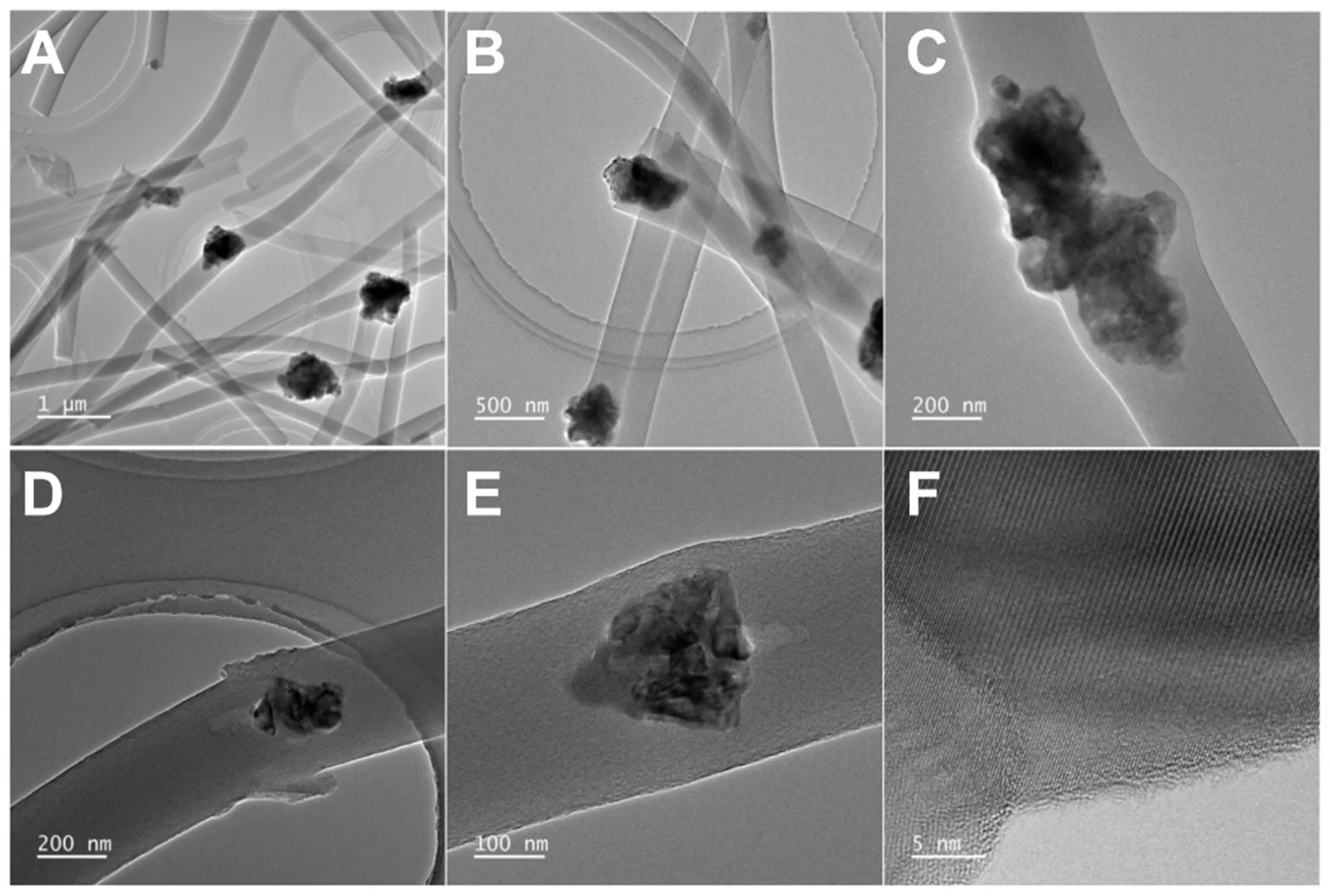
(**A**) Low-magnification TEM overview of the CNF@TiN composite nanofibers; (**B**) TEM image of TiN particles dispersed on the carbon nanofibers; (**C**) TEM image of TiN particles supported on a carbon nanofiber; (**D**) TEM image of a TiN particle anchored on an individual carbon nanofiber; (**E**) TEM image of a TiN particle in intimate contact with a carbon nanofiber; (**F**) HRTEM image showing clear lattice fringes of the crystalline TiN particle.

Figure 4 presents TEM and HRTEM images of the CNF@TiN composite nanofibers at different magnifications. As shown in Figure 4A–E, the one-dimensional carbon nanofibers interweave to form a continuous network framework, while TiN particles are dispersed and supported on the carbon nanofiber surfaces or at fiber junctions, without obvious large-scale detachment or severe aggregation. The continuous CNF framework can provide structural support for TiN particles and establish interconnected electron-transport pathways. Clear and continuous lattice fringes are observed in Figure 4F, indicating that the supported particles possess good crystallinity, in agreement with the XRD results described above. The intimate contact between the TiN particles and the conductive CNF framework is expected to ensure efficient electron supply to the TiN/polysulfide interface during cycling [28,30]. This composite architecture simultaneously provides a continuous carbon network and exposed TiN interfaces, establishing a structural basis for polysulfide confinement and interfacial reactions.

**Figure 5:**
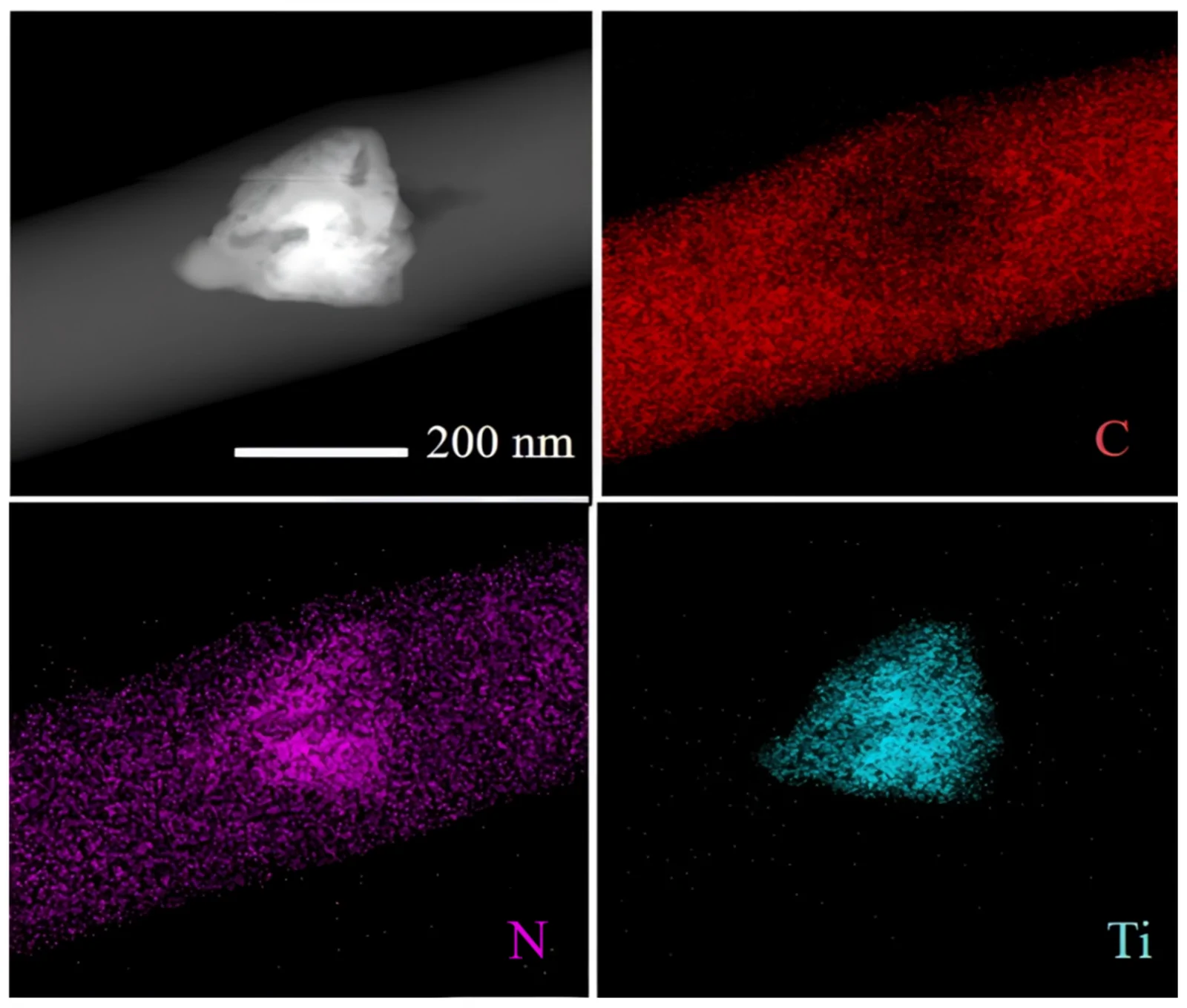
TEM image and corresponding EDS elemental maps of the CNF@TiN composite nanofibers.

Figure 5 further presents the TEM image and corresponding EDS elemental maps of the CNF@TiN sample. The C signal is continuously distributed along the nanofibers, outlining the carbon nanofiber framework. The Ti signal is mainly concentrated in the particulate regions, while the N signal shows good spatial overlap with Ti in the corresponding regions. Combined with the XRD and XPS results described above, these particles can be identified primarily as TiN [8,17]. The elemental maps correspond well with the TEM morphology, further demonstrating that TiN particles are supported within the CNF network and form a relatively uniform composite structure.

Based on the above XPS results and the microscopic observations, the interfacial interaction between the carbon scaffold and the TiN particles can be discussed more specifically. In the HRTEM images (Figure 4F), the lattice fringes of the crystalline TiN particles extend to the particle surface in direct contact with the carbon matrix, and no thick amorphous intermediate layer is resolved, which suggests an intimate solid–solid contact between the two phases. The N- and O-containing functional groups identified on the PAN-derived carbon surface (Figure S1) are expected to act as heterogeneous anchoring sites that favor the adhesion and uniform dispersion of the polar TiN particles during pre-oxidation and carbonization, consistent with the absence of large-scale particle detachment or aggregation in the SEM and TEM observations. Moreover, because TiN is metallic-like and the CNF network is electronically conductive, the intimate CNF–TiN contact establishes low-resistance electron pathways from the carbon scaffold to the polar TiN surface, where polysulfide adsorption and conversion take place [27,28,30]. This interfacial coupling between the defective, heteroatom-functionalized carbon and the polar nitride is therefore regarded as a key structural factor underlying the improved redox kinetics and cycling stability discussed in Section 3.2. It should be noted that the present data do not allow the interfacial bonding to be resolved at the atomic level; the discussion above is based on the combined XPS, HRTEM, and EDS evidence and on analogy with related TiN/carbon systems [27,30].

The porous structures of the interlayers were characterized by N2 adsorption–desorption measurements (Figure S3 and Table S1). The pristine CNF membrane exhibits a BET specific surface area of 18.4 m^2^·g^−1^ and a total pore volume of 0.112 cm^3^·g^−1^, and the corresponding values for the CNF@TiN composite membrane are 13.3 m^2^·g^−1^ and 0.026 cm^3^·g^−1^, respectively. Both isotherms display type-IV characteristics with H3-type hysteresis loops according to the IUPAC classification [35], indicating the presence of mesopores together with inter-fiber macroporous voids, and the pore-size distributions are broad, spanning approximately 3–100 nm (Figure S3b). The moderate decrease in surface area and pore volume upon TiN incorporation is consistent with the partial occupation of the inter-fiber void space by the dense TiN particles, while the membrane retains a porous, interwoven fibrous architecture. Such a porous architecture is expected to facilitate electrolyte infiltration and ion transport through the interlayer while providing accessible surfaces for polysulfide interaction [22,26].

### 2.2. Electrochemical Performance

Unless otherwise specified, all electrochemical data presented in this section were obtained with a sulfur areal loading of approximately 1.8 mg·cm^−2^, an electrolyte-to-sulfur ratio of 25 μL·mg^−1^, and a voltage window of 1.6–2.8 V vs. Li^+^/Li; specific capacities are calculated based on the mass of sulfur, and at least three cells were tested for each configuration (see Section 3.7 for full details).

**Figure 6:**
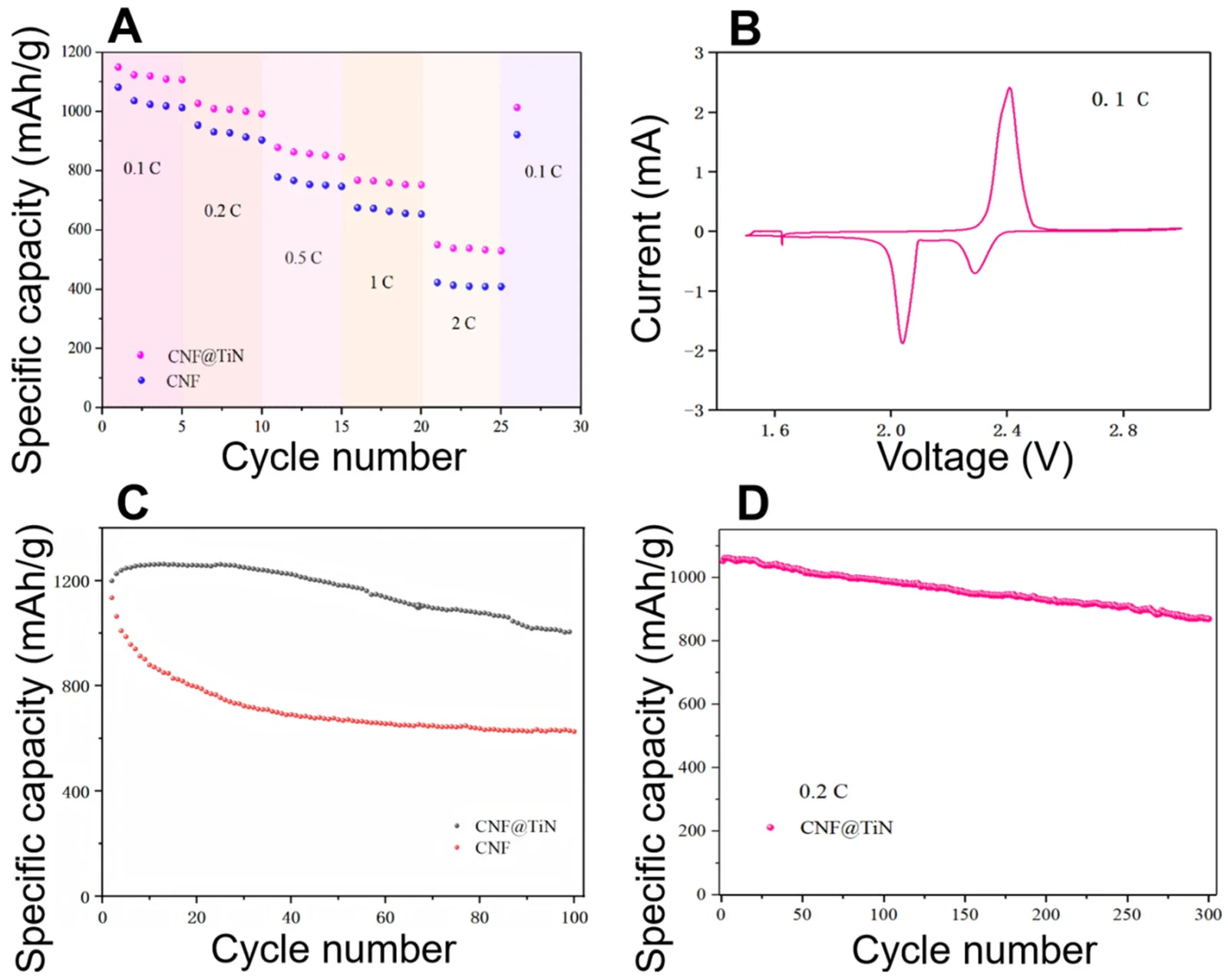
(**A**) Rate capability of the cells with the CNF and CNF@TiN interlayers; (**B**) CV curve of the cell with the CNF@TiN interlayer; (**C**) comparison of cycling performance at 0.1 C for cells with CNF and CNF@TiN interlayers; (**D**) long-term cycling performance of the cell with the CNF@TiN interlayer at 0.2 C.

Figure 6A shows the rate capability of the cell with the CNF@TiN interlayer over the range of 0.1–2 C. At 0.1 C, the cell delivers an initial discharge specific capacity of approximately 1149 mAh·g^−1^. When the rate is sequentially increased to 0.2, 0.5, 1, and 2 C, reversible discharge capacities of approximately 1029, 878, 767, and 550 mAh·g^−1^ are maintained, respectively. When the rate is returned from 2 C to 0.1 C, the discharge specific capacity recovers to approximately 1013 mAh·g^−1^, indicating good capacity recovery after high-rate cycling. The capacity recovery suggests that the interwoven CNF network retains its structural integrity and that the TiN/polysulfide interfaces remain accessible after high-rate operation. The gradual capacity decrease with increasing current density is commonly observed for interlayer-modified Li–S cells and is mainly associated with the finite ionic-transport rate through the interlayer and incomplete sulfur utilization at high rates [22,30]. A quantitative comparison with representative reported systems is provided in Table 1.

Figure 6B shows the cyclic voltammetry (CV) curve of the cell with the CNF@TiN interlayer. Within the voltage window of 1.6–2.8 V, two distinct reduction peaks are observed during the cathodic scan, corresponding to the reduction of S8 to long-chain lithium polysulfides and the subsequent conversion of long-chain polysulfides to Li_2_S_2_/Li_2_S, respectively [15,17,27]. During the anodic scan, an oxidation peak appears at approximately 2.45 V, corresponding to the reverse oxidation of lower-order sulfides to higher-order polysulfides and ultimately to elemental sulfur [27,28]. The well-defined redox peaks indicate that the CNF@TiN interlayer system can maintain the characteristic multistep sulfur redox reactions. The relatively symmetric redox peak shapes further suggest reversible sulfur conversion on the CNF@TiN interlayer [15].

Figure 6C compares the cycling performance of cells with CNF and CNF@TiN interlayers at 0.1 C. The CNF@TiN cell exhibits an initial discharge specific capacity of approximately 1205 mAh·g^−1^. After an initial activation period, the capacity gradually stabilizes and remains at approximately 1010 mAh·g^−1^ after 100 cycles (≈83% of the first-cycle capacity), whereas the CNF cell retains only about 630 mAh·g^−1^ (≈58% of its initial capacity). In comparison, the cell with the CNF interlayer shows a lower initial capacity and more pronounced capacity fading. These results indicate that the incorporation of TiN into the conductive CNF network enhances interfacial regulation of soluble polysulfides, thereby mitigating active-material loss and improving cycling stability. The improvement over the CNF-only interlayer is attributed to the additional polar adsorption and interfacial regulation provided by the uniformly dispersed TiN particles, consistent with the behavior reported for other TiN/carbon systems [19,27,28]. Notably, the magnitude of this improvement over the pristine CNF interlayer is comparable to the enhancement reported when polar nitride or oxide phases are introduced into other electrospun carbon-based interlayers [26,30].

Figure 6D shows the long-term cycling performance of the cell with the CNF@TiN interlayer at 0.2 C. The initial discharge specific capacity is approximately 1065 mAh·g^−1^, and approximately 875 mAh·g^−1^ is retained after 300 cycles, corresponding to a capacity retention of 82.2% and an average capacity decay of approximately 0.059% per cycle. The capacity evolves smoothly throughout cycling without an obvious abrupt drop. The capacity variation during the initial cycles may be associated with gradual electrolyte wetting, progressive participation of active sulfur in the reaction, and adjustment of the interfacial state. Thereafter, the rate of capacity fading becomes slower, indicating that the system can maintain relatively stable reversible reactions. The continuous CNF network provides electron-transport pathways and structural support, while the polar TiN interface helps restrict the migration of soluble polysulfides and facilitates their interfacial conversion. The combination of these complementary functions is therefore beneficial for long-term cycling stability. Notably, this cycling stability is competitive with or superior to most reported TiN-based and electrospun carbon interlayers tested under comparable conditions, as summarized in Table 1.

As summarized in Table 1, the present CNF@TiN interlayer delivers a capacity retention of 82.2% after 300 cycles at 0.2 C, which is competitive with or superior to most reported TiN-based interlayers and electrospun carbon interlayers tested under comparable conditions [15,17,27,28,29,30]. This good cycling stability is ascribed to the combination of the continuous three-dimensional CNF network—which retards polysulfide diffusion and provides electron-transport pathways—with the uniformly exposed polar TiN interfaces, whose presence is consistent with strengthened polysulfide adsorption and facilitated conversion [26,28]. The initial discharge capacity (≈1205 mAh·g^−1^ at 0.1 C) is close to the values reported for highly conductive TiN/carbon systems [17,27], indicating high sulfur utilization. In contrast, the capacity at 2 C (≈550 mAh·g^−1^) is moderate compared with the best catalytic systems [28]; this difference is tentatively attributed to the limited accessibility of TiN sites embedded within the fibers and to the increased ionic-transport resistance of the relatively dense interlayer at high current densities. Further optimization of the TiN particle size, loading, and interlayer porosity is expected to improve the high-rate performance. Finally, it should be noted that the present data were obtained under moderate conditions (sulfur loading of approximately 1.8 mg·cm^−2^ and E/S = 25 μL·mg^−1^), which are less demanding than those of some reported systems (e.g., 3.8 mg·cm^−2^ sulfur loading and 10 μL·mg^−1^ electrolyte in ref. [30]). The results therefore demonstrate the effectiveness of the interlayer at the materials level; validation under high-loading, lean-electrolyte conditions remains to be carried out.

**Figure 7:**
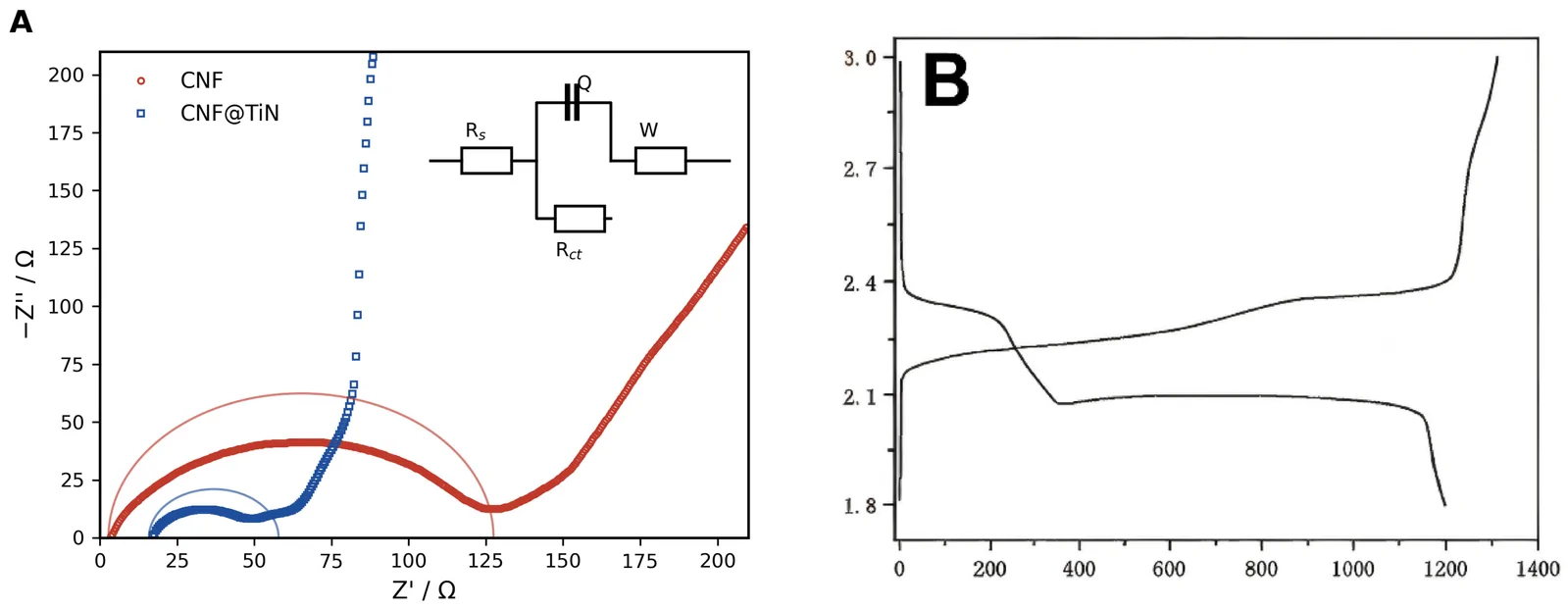
(**A**) Nyquist plots of the cells with the CNF and CNF@TiN interlayers (inset: equivalent circuit used for fitting); (**B**) first-cycle galvanostatic charge–discharge profile of the cell with the CNF@TiN interlayer at 0.1 C.

Figure 7A shows the electrochemical impedance spectra (EIS) of the cells with the CNF and CNF@TiN interlayers. The Nyquist plot mainly consists of a high-frequency ohmic intercept, a semicircle in the medium-to-high-frequency region associated with charge transfer, and an inclined diffusion tail in the low-frequency region, reflecting the ohmic resistance, interfacial charge-transfer process, and ion diffusion behavior of the cell, respectively [22,30]. This impedance response suggests relatively continuous interfacial transport characteristics in the CNF@TiN interlayer system. Together with the rate and cycling results, these observations suggest that the conductive CNF network and TiN interface provide favorable conditions for electron/ion transport and reversible sulfur-species conversion. In particular, the moderate semicircle indicates an accessible charge-transfer process at the interlayer/electrolyte interface, consistent with the conductive CNF network and polar TiN interface facilitating electron transfer and polysulfide conversion [15,28]. For quantitative comparison, the impedance spectra of the CNF and CNF@TiN cells, measured under identical conditions, were fitted using the equivalent circuit shown in the inset of Figure 7A (Rs(Q(RctW)), where Rs denotes the ohmic resistance, Rct the charge-transfer resistance, Q a constant-phase element, and W the Warburg diffusion element). The fitted charge-transfer resistance of the CNF@TiN cell (Rct = 42 Ω) is lower than that of the CNF control cell (Rct = 125 Ω). The ohmic resistance of the CNF@TiN cell (Rs ≈ 16 Ω) is moderately higher than that of the CNF cell (≈3 Ω), which can be attributed to the additional thickness and contact resistance introduced by the composite interlayer; the relative fitting errors are below 5%. This quantitative comparison supports assigning the reduced polarization of the CNF@TiN cell to the presence of the TiN component.

Figure 7B shows the first-cycle galvanostatic charge–discharge profile of the cell with the CNF@TiN interlayer at 0.1 C. Two characteristic discharge plateaus are observed at approximately 2.35 and 2.10 V, corresponding to the stepwise reduction of S8 to long-chain lithium polysulfides and then of long-chain polysulfides to Li_2_S_2_/Li_2_S, respectively [17,27]. The charging plateau is mainly located near 2.4 V and corresponds to the oxidation of lower-order sulfides to higher-order polysulfides and elemental sulfur [15]. The well-defined two-step discharge plateaus are consistent with the CV results, confirming that the system undergoes the characteristic multistep sulfur redox conversion.

## 3. Materials and Methods

### 3.1. Preparation of the Pristine CNF Interlayer

Polyacrylonitrile (PAN, 0.5 g; Shanghai Aladdin Biochemical Technology Co., Ltd., Shanghai, China) was added to a serum bottle, followed by 5 mL of N,N-dimethylformamide (DMF, Sinopharm Chemical Reagent Co., Ltd., Shanghai, China). The bottle was sealed and the mixture was stirred on a thermostatic magnetic stirrer until a homogeneous transparent solution was obtained. Electrospinning was then performed at an applied voltage of 15 kV and a solution feeding rate of 1.0 mL·h^−1^ for approximately 4 h. The collected PAN nanofiber membrane was dried in an oven at 80 °C. The dried membrane was cut into pieces of approximately 7 cm × 3 cm and clamped between two graphite plates to maintain flatness before thermal treatment in a tube furnace (Hefei Kejing Materials Technology Co., Ltd., Hefei, China). Pre-oxidation was first conducted in air by heating to 250 °C at a rate of 2 °C·min^−1^. Subsequently, carbonization was performed under an Ar atmosphere by heating to 800 °C at 2 °C·min^−1^ and holding for 2 h. After naturally cooling to room temperature, a carbon nanofiber (CNF) interlayer was obtained.

### 3.2. Preparation of TiN Powder

A certain amount of commercial nanosized TiO_2_ powder (Shanghai Aladdin Biochemical Technology Co., Ltd., Shanghai, China) was dispersed in a 10 mol·L^−1^ NaOH (Sinopharm Chemical Reagent Co., Ltd., Shanghai, China) aqueous solution and magnetically stirred thoroughly to form a homogeneous suspension. The suspension was transferred into a Teflon-lined stainless-steel autoclave (Gongyi Yuhua Instrument Co., Ltd., Gongyi, China) and hydrothermally treated at 180 °C for 24 h. After the reaction, the autoclave was naturally cooled to room temperature, and the white precipitate was collected by centrifugation. The product was repeatedly washed with deionized water and 1 mol·L^−1^ HCl (Sinopharm Chemical Reagent Co., Ltd., Shanghai, China) solution until the supernatant became neutral to remove residual Na^+^. After drying at 80 °C, a titanate nanofiber precursor was obtained. The precursor was placed in a corundum boat and transferred into a horizontal tube furnace, heated to 800 °C at 5 °C·min^−1^ under an NH_3_ atmosphere, and held for 2 h for high-temperature nitridation. The furnace was then naturally cooled to room temperature to obtain black TiN powder.

### 3.3. Preparation of the CNF@TiN Composite Interlayer

Polyacrylonitrile (PAN, 0.5 g) was added to a 25 mL serum bottle containing 5 mL of DMF and stirred in an oil bath at 70 °C for 1 h to completely dissolve the PAN. Subsequently, 2 mmol of pre-prepared TiN particles was added, and the mixture was stirred at the same temperature for another 6 h to obtain a uniformly dispersed PAN/TiN spinning solution. The solution was transferred into a 10 mL plastic syringe fitted with a 23-gauge blunt metal needle and mounted on the electrospinning apparatus. The electrospinning voltage was set to 18 kV, the needle-to-collector distance was 15 cm, the rotating drum speed was 500 rpm, and the solution feeding rate was 1.0 mL·h^−1^. After a sufficient amount of fibrous mat had been collected, it was peeled from the drum and dried overnight at 70 °C. The dried sample was cut to the desired dimensions and thermally treated using the same pre-oxidation and carbonization procedure as that used for the pristine CNF interlayer. After naturally cooling to room temperature, the CNF@TiN composite nanofiber interlayer was obtained. Based on the feed composition (2 mmol TiN per 0.5 g PAN, corresponding to approximately 20 wt% of the total PAN/TiN precursor mass), the TiN mass fraction in the final carbonized composite is approximately 33 wt% when estimated from the feed composition assuming a PAN-to-carbon conversion yield of about 50% during carbonization; thermogravimetric quantification will be carried out in future work. The self-standing composite membrane has a thickness of approximately 50–100 μm (depending on the electrospinning duration); the TiN particle diameters observed by SEM are mainly in the range of 30–100 nm, with a number-averaged mean diameter of approximately 59 nm (Figure S4).

### 3.4. Preparation of the Sulfur Cathode

Preparation of the S/CNT composite: Sublimed sulfur (Shanghai Aladdin Biochemical Technology Co., Ltd., Shanghai, China) and commercial carbon nanotubes (CNTs, Shanghai Aladdin Biochemical Technology Co., Ltd., Shanghai, China) were mixed at a mass ratio of 70:30 in an agate mortar and thoroughly ground for 1 h. The mixture was then transferred to a sealed glass container and maintained at 155 °C for 12 h, allowing molten sulfur to sufficiently infiltrate and load onto the CNT surfaces and pores. After naturally cooling to room temperature, an S/CNT composite with a sulfur content of 70 wt% was obtained.

Preparation of the cathode disks: The above S/CNT composite, acetylene black (Sinopharm Chemical Reagent Co., Ltd., Shanghai, China), and poly(vinylidene fluoride) (PVDF, Shanghai Aladdin Biochemical Technology Co., Ltd., Shanghai, China) were mixed at a mass ratio of 8:1:1. An appropriate amount of N-methyl-2-pyrrolidone (NMP, Sinopharm Chemical Reagent Co., Ltd., Shanghai, China) was added, and the mixture was thoroughly stirred to form a homogeneous slurry. The slurry was uniformly coated onto one side of 20 μm-thick aluminum foil (Sinopharm Chemical Reagent Co., Ltd., Shanghai, China) using a doctor blade and vacuum-dried at 60 °C for 12 h to remove the solvent. After drying, the electrode sheet was punched into circular disks with a diameter of 12 mm. Based on mass measurements, the sulfur areal loading was approximately 1.8 mg·cm^−2^.

Preparation of high-sulfur-loading cathodes: An S/CNT composite with a sulfur content of 80 wt% was first prepared using the same method described above. The S/CNT composite, acetylene black, and PVDF were then mixed at a mass ratio of 85:10:5, and an appropriate amount of NMP was added to form a homogeneous slurry. Cathode disks were prepared using the same coating, vacuum-drying, and punching procedures. By adjusting the coating thickness, cathodes with sulfur areal loadings of approximately 2–5 mg·cm^−2^ were obtained. It should be noted that these high-loading cathodes were prepared to demonstrate the compatibility of the cathode-fabrication procedure with higher sulfur loadings; systematic high-loading electrochemical evaluation is beyond the scope of the present study and will be reported in future work.

### 3.5. Cell Assembly

CR2032 coin cells (Shenzhen Neware Technology Co., Ltd., Shenzhen, China) were assembled in an Ar-filled glovebox (M. Braun Inertgas-Systeme GmbH, Garching, Germany) in which both H_2_O and O_2_ concentrations were below 0.1 ppm. Lithium-metal foil (Sinopharm Chemical Reagent Co., Ltd., Shanghai, China), S/CNT-coated aluminum foil, and Celgard 2400 (Celgard LLC., Charlotte, NC, USA) were used as the anode, cathode, and separator, respectively. The electrolyte consisted of 1,2-dimethoxyethane (DME, Suzhou Duoduo Chemical Reagent Co., Ltd., Suzhou, China) and 1,3-dioxolane (DOL, Suzhou Duoduo Chemical Reagent Co., Ltd., Suzhou, China) at a volume ratio of 1:1 and contained 1.0 M lithium bis(trifluoromethanesulfonyl)imide (LiTFSI, Suzhou Duoduo Chemical Reagent Co., Ltd., Suzhou, China) and 1.0 wt% LiNO_3_ (Suzhou Duoduo Chemical Reagent Co., Ltd., Suzhou, China). The electrolyte-to-sulfur (E/S) ratio was 25 μL·mg^−1^. After assembly, the cells were allowed to rest for 12 h for activation before subsequent electrochemical testing.

### 3.6. Materials Characterization

The crystal structure of the prepared TiN powder was characterized by X-ray diffraction (XRD) on a Bruker D8 Advance diffractometer (Bruker AXS, Karlsruhe, Germany) using Cu Kα radiation (λ = 1.5406 Å) operated at 40 kV and 40 mA. Diffraction data were collected over a 2θ range of 10–80° with a step size of 0.02° and a scan rate of 0.5°·min^−1^. Phase identification was performed by comparison with the standard PDF cards from the ICDD/JCPDS database [36] and with values reported in the literature. The surface chemical states and elemental bonding configurations were analyzed by X-ray photoelectron spectroscopy (XPS) on a Kratos spectrometer (Kratos Analytical, Manchester, UK) equipped with a monochromatic Al Kα source (hν = 1486.6 eV). Survey and high-resolution spectra were collected with pass energies of 160 eV and 40 eV, respectively (step sizes of 1.0 eV and 0.1 eV). The binding-energy scale was calibrated against the adventitious carbon C 1s peak at 284.8 eV, which was also used for charge correction; for the sputter-etched TiN powder, the Ti–N 2p3/2 component at 455.0 eV was used as the internal reference. Peak deconvolution was performed using a Shirley-type background and mixed Gaussian–Lorentzian line shapes; for the Ti 2p region the spin–orbit splitting was fixed at 5.7 eV, the 2p3/2:2p1/2 area ratio was fixed at 2:1, and the 2p1/2 component was allowed a slightly larger FWHM than the 2p3/2 component to account for Coster–Kronig broadening. SEM analysis was performed using a JEOL JSM-7900F (JEOL Ltd., Tokyo, Japan) at an accelerating voltage of 3–10 kV; the membranes were mounted on conductive carbon tape prior to imaging. Transmission electron microscopy (TEM), high-resolution TEM (HRTEM), and energy-dispersive X-ray spectroscopy (EDS) elemental mapping were performed on a JEOL JEM-F200 transmission electron microscope (JEOL Ltd., Tokyo, Japan) operated at 200 kV, equipped with an Oxford Instruments Ultim Max 80 (Oxford Instruments, Abingdon, UK) energy-dispersive X-ray spectrometer. For TEM analysis, the composite nanofibers were dispersed in ethanol by ultrasonication and drop-cast onto copper grids covered with a lacey carbon support film. EDS elemental mapping was employed to analyze the spatial distribution of C, N, and Ti within the composite nanofibers. Additional XPS measurements were performed on the CNF@TiN composite interlayer (high-resolution Ti 2p, N 1s, O 1s, and C 1s spectra; Figure S1) and on the as-prepared TiN powder (survey scan and high-resolution Ti 2p, N 1s, O 1s, and C 1s spectra acquired after Ar+ sputter etching for 430 s to expose the subsurface beneath the native oxide layer; Figure S2), using the same instrument, calibration, and fitting procedure described above. Nitrogen adsorption–desorption isotherms of the CNF and CNF@TiN membranes were collected at 77 K on a Micromeritics ASAP 2460 analyzer (Micromeritics Instrument Corporation, Norcross, GA, USA) after degassing under vacuum at 180 °C for 8 h; the specific surface area was calculated by the Brunauer–Emmett–Teller (BET) method, and the pore-size distribution was derived from the desorption branch by the Barrett–Joyner–Halenda (BJH) method (Supplementary Material, Figure S3).

### 3.7. Electrochemical Measurements

Electrochemical performance was evaluated using CR2032-type coin cells assembled as described in Section 3.5. The two-electrode cell consisted of a lithium-metal foil anode, a Celgard 2400 polypropylene separator, the CNF or CNF@TiN interlayer (placed between the separator and the cathode, facing the cathode), and an S/CNT composite cathode (12 mm in diameter; sulfur areal loading of approximately 1.8 mg·cm^−2^ unless otherwise stated). Unless specified otherwise, the cathode slurry composition was S/CNT composite:acetylene black:PVDF = 8:1:1 (*w*/*w*/*w*), and the sulfur content in the S/CNT composite was 70 wt%. The electrolyte was 1.0 M LiTFSI in DME/DOL (1:1, *v*/*v*) containing 1.0 wt% LiNO_3_, and the electrolyte-to-sulfur (E/S) ratio was 25 μL·mg^−1^. All electrochemical tests were performed at room temperature (25 ± 2 °C). After assembly, the cells were rested for 12 h before testing. Galvanostatic charge–discharge cycling and rate-capability tests were conducted on a Neware CT-4008 battery test system (Shenzhen Neware Technology Co., Ltd., Shenzhen, China). Cyclic voltammetry (CV) was performed on a CHI660E (Shanghai Chenhua Instrument Co., Ltd., Shanghai, China) electrochemical workstation at a scan rate of 0.1 mV·s^−1^ for three consecutive cycles. Electrochemical impedance spectroscopy (EIS) was recorded at the open-circuit potential of freshly assembled cells over a frequency range of 0.01 Hz–100 kHz with an AC perturbation amplitude of 5 mV. For each cell configuration, at least three cells were assembled and tested, and the data presented are from representative cells. All stated potentials (e.g., the testing window of 1.6–2.8 V) refer to the cell voltage versus the Li^+^/Li redox couple, i.e., the potential of the sulfur cathode referenced to the lithium-metal counter electrode in the two-electrode coin-cell configuration; specific capacities were calculated based on the mass of sulfur in the cathode.

## 4. Conclusions

In this study, TiN powder was prepared by high-temperature nitridation, and a CNF@TiN composite nanofiber interlayer was subsequently constructed through electrospinning, pre-oxidation, and carbonization and applied in Li-S batteries. Structural characterization showed that TiN particles were relatively uniformly supported within the three-dimensional interconnected CNF network while maintaining good crystallinity. XPS analyses further showed that the PAN-derived carbon scaffold is N/O-functionalized and moderately defective, that the TiN particles retain their nitride character with only a thin surface-oxidized layer, and that the two phases form an intimate interfacial contact. The cell equipped with the CNF@TiN interlayer delivered an initial discharge specific capacity of approximately 1205 mAh·g^−1^ at 0.1 C and maintained a reversible capacity of approximately 1010 mAh·g^−1^ after 100 cycles. At 0.2 C, the initial discharge specific capacity was approximately 1065 mAh·g^−1^, and approximately 875 mAh·g^−1^ remained after 300 cycles, corresponding to a capacity retention of 82.2% and an average capacity decay of approximately 0.059% per cycle. The rate capability, CV, EIS, and galvanostatic charge–discharge results collectively indicate that the CNF@TiN composite interlayer is beneficial for maintaining the reversibility of sulfur redox reactions and improving interfacial transport behavior. These performance advantages mainly arise from the physical confinement, electron-transport, and structural-support functions of the three-dimensional CNF network, together with the polar TiN interface, whose presence is consistent with restricted polysulfide migration and facilitated interfacial conversion. These results indicate that CNF@TiN composite nanofibers are promising functional interlayer materials for Li-S batteries under the tested conditions; validation under high-loading and lean-electrolyte conditions will be pursued in future work.

## Figures and Tables

**Table 1:** Comparison of the cycling performance of the CNF@TiN composite interlayer with representative TiN-based and carbon-based interlayers/hosts for Li–S batteries reported in the literature.

Interlayer/Host	S Loading (mg·cm^−2^)	Discharge Capacity (mAh∙g^−1^) and Rate	Cycles	Capacity Retention	Ref.
CNF@TiN composite interlayer	1.8	1065 (0.2 C)	300	82.2%	This work
TiN-based membrane interlayer	1.1–1.5	851.4 (5 C)	200	94.8%	[15]
Mesoporous TiN host	1.0	988 (0.5 C)	500	65.2%	[17]
Coaxial CNTs@TiN–TiO_2_ sponge	15	900 (2 C)	500	85%	[27]
TiN–VN heterostructure framework	5.6	876 (2 C)	>600	69%	[28]
2D TiN nanosheet separator coating	1.2	854 (1 C)	1000	54.2%	[29]
Cu-embedded CNF/TiN interlayer	1.5–2.0	1340 (0.5 C)	300	46.5%	[30]

## Data Availability

The original contributions presented in this study are included in the article/Supplementary Material. Further inquiries can be directed to the corresponding authors.

## Supplementary Materials

The following supporting information can be downloaded at https://www.mdpi.com/article/10.3390/molecules31203628/s1, Figure S1: High-resolution XPS spectra of the CNF@TiN composite interlayer with peak deconvolution: (a) C 1s, (b) N 1s, (c) O 1s, and (d) Ti 2p. All spectra were charge-corrected against the C–C/C=C component at 284.8 eV and fitted with Shirley backgrounds. The Ti 2p spectrum in (d) is the same dataset as Figure 2B of the main text; Figure S2: XPS characterization of the as-prepared TiN powder after Ar+ sputter etching for 430 s to expose the subsurface beneath the native oxide layer: (a) survey spectrum (photoelectron lines are labeled; O KLL and Ti LMM Auger features are also indicated), and high-resolution (b) Ti 2p, (c) N 1s, and (d) O 1s spectra with peak deconvolution. No impurity elements (e.g., residual Na from the synthesis) were detected within the XPS detection limit; Figure S3: (a) N2 adsorption–desorption isotherms (77 K) and (b) BJH desorption pore-size distributions of the pristine CNF membrane and the CNF@TiN composite membrane; Figure S4: (a) SEM image of the as-prepared TiN powder and (b) the corresponding particle-size distribution histogram obtained from statistical analysis of the SEM images (n = 114 particles; number-averaged mean diameter ≈ 59 nm, d10 ≈ 28 nm, d90 ≈ 96 nm); Table S1: Summary of the BET and BJH results for the CNF and CNF@TiN membranes.
