# Supplementary material for: Electrospun Carbon Nanofiber–TiN Composite Interlayers for Improved Cycling Stability and Rate Capability of Lithium–Sulfur Batteries
Source: Molecules. 2026 Oct 12;31(20):3628. doi: 10.3390/molecules31203628 (PMC13707449; doi:10.3390/molecules31203628)
Supplement: Supplementary file 1 [file molecules-31-03628-s001.zip › molecules-4574224-supplementary.pdf]

Supplementary Materials

Electrospun Carbon Nanofiber–TiN Composite Interlayers for Improved Cycling Stability and Rate Capability of Lithium–Sulfur Batteries

Chaoqun Niu [1], Huan Liu [2,3,*] and Zisheng Chao [4,*]

[1] School of Physics and Electronic Science, Changsha University of Science & Technology, Changsha 410114, China; chaoqunniu@yeah.net
[2] Key Laboratory of Energy Conversion Photoelectronic Functional Materials of Jiangxi Education Institutes, School of Mathematics and Physics, Jinggangshan University, Ji'an 343009, China
[3] Shinawatra University, 99, Bang Toei, Sam Khok District, Pathum Thani 12160, Thailand
[4] School of Materials Science and Engineering, Changsha University of Science & Technology, Changsha 410114, China
* Correspondence: liu_huan8899@163.com (H.L.); zschao@hnu.edu.cn (Z.C.)

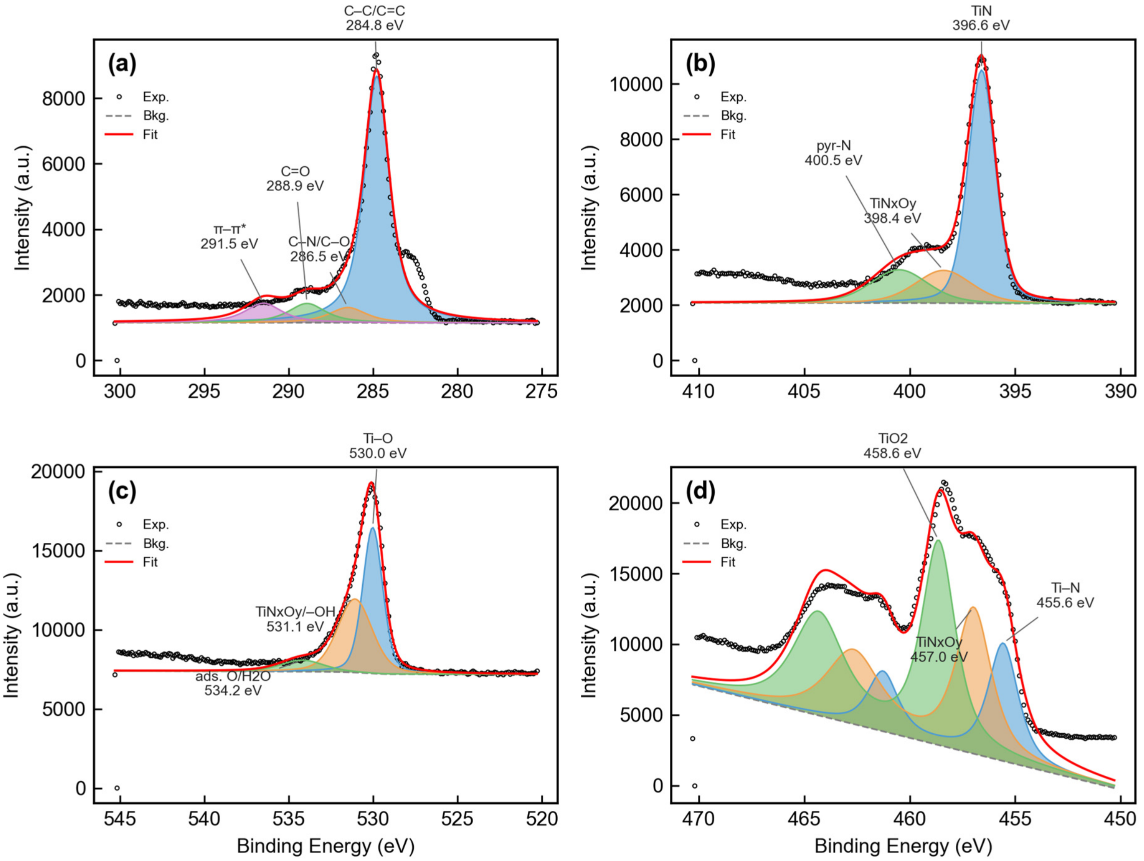

Figure S1. High-resolution XPS spectra of the CNF@TiN composite interlayer with peak deconvolution: (a) C 1s, (b) N 1s, (c) O 1s, and (d) Ti 2p. All spectra were charge-corrected against the C–C/C=C component at 284.8 eV and fitted with Shirley backgrounds. The Ti 2p spectrum in (d) is the same dataset as Figure 2B of the main text.

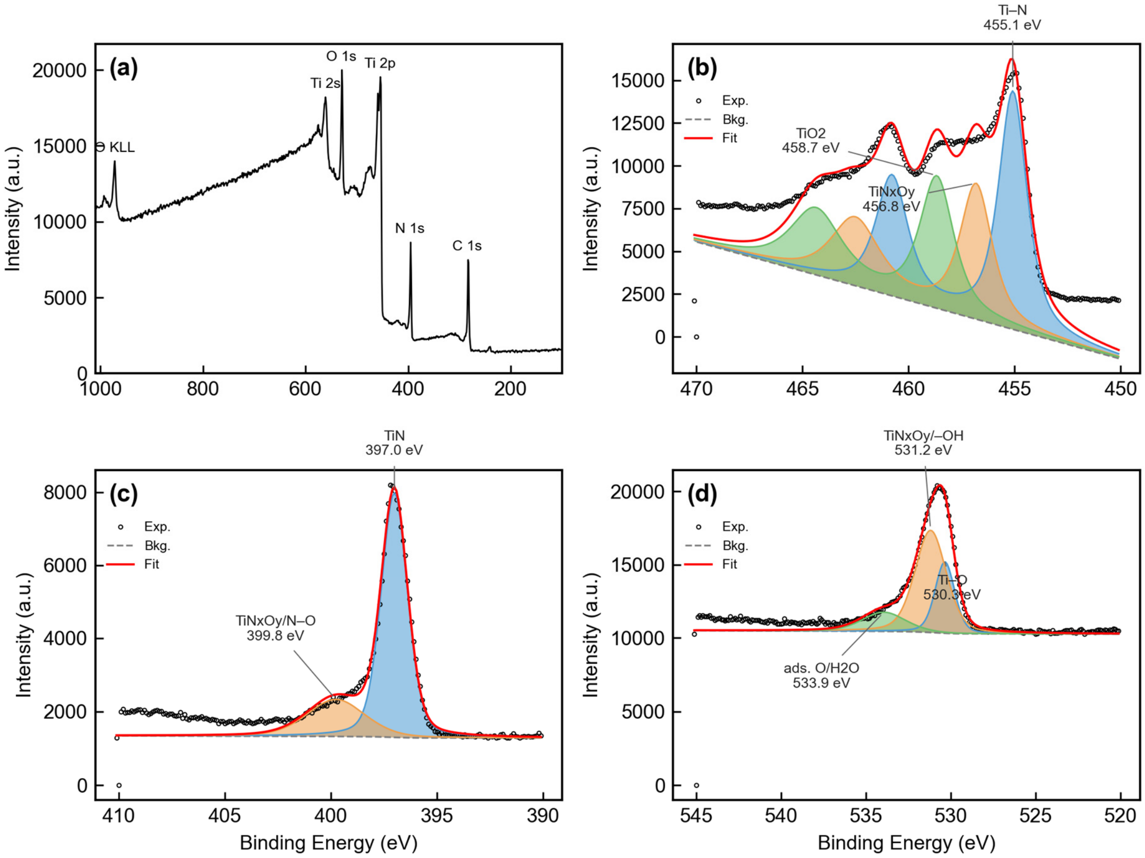

Figure S2. XPS characterization of the as-prepared TiN powder after Ar+ sputter etching for 430 s to expose the subsurface beneath the native oxide layer: (a) survey spectrum (photoelectron lines are labeled; O KLL and Ti LMM Auger features are also indicated), and high-resolution (b) Ti 2p, (c) N 1s, and (d) O 1s spectra with peak deconvolution. No impurity elements (e.g., residual Na from the synthesis) were detected within the XPS detection limit.

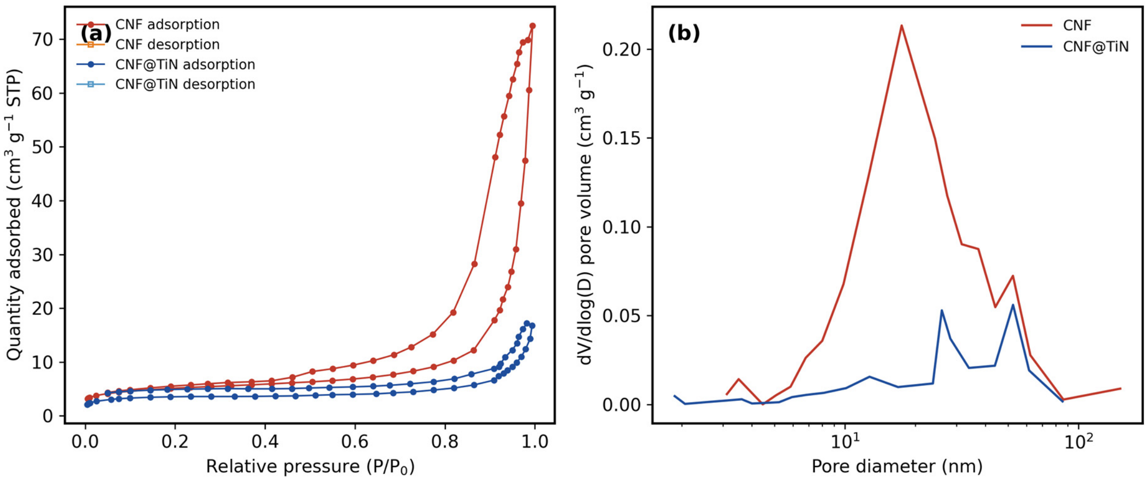

Figure S3. (a) N2 adsorption–desorption isotherms (77 K) and (b) BJH desorption pore-size distributions of the pristine CNF membrane and the CNF@TiN composite membrane.

Table S1. Summary of the BET and BJH results for the CNF and CNF@TiN membranes.

**Sample**	**BET surface area (m2 g−1)**	**Total pore volume (cm3 g−1)**	**Average pore diameter (nm, 4V/A by BET)**	**t-Plot micropore area (m2 g−1)**
CNF	18.38	0.112	24.4	7.80
CNF@TiN	13.27	0.026	7.8	7.71

Note: Nitrogen adsorption–desorption isotherms were collected at 77 K on a Micromeritics ASAP 2460 analyzer after degassing under vacuum at 180 °C for 8 h. The specific surface area was calculated by the BET method, and the pore-size distribution was derived from the desorption branch by the BJH method.

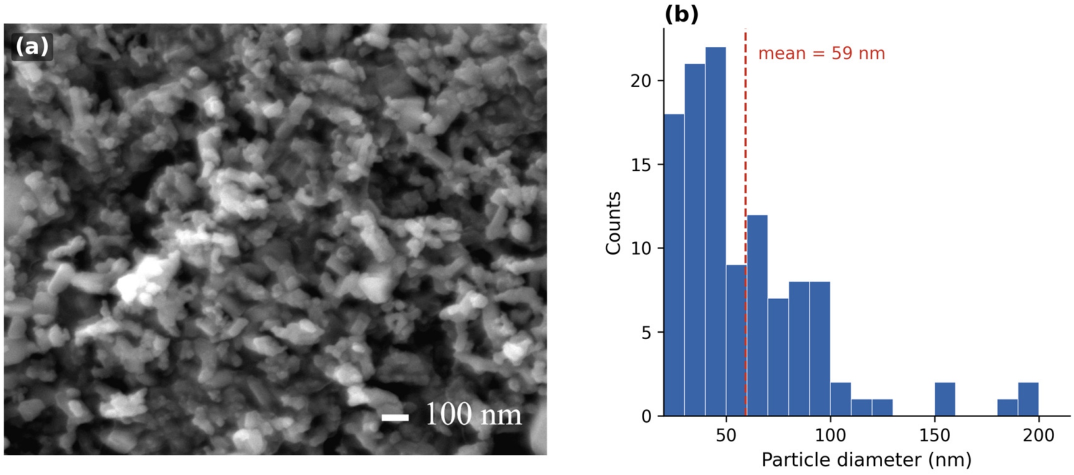

Figure S4. (a) SEM image of the as-prepared TiN powder and (b) the corresponding particle-size distribution histogram obtained from statistical analysis of the SEM images (n = 114 particles; number-averaged mean diameter ≈ 59 nm, d10 ≈ 28 nm, d90 ≈ 96 nm).
